# Round Randomized Learning Vector Quantization for Brain Tumor Imaging

**DOI:** 10.1155/2016/8603609

**Published:** 2016-07-18

**Authors:** Siti Norul Huda Sheikh Abdullah, Farah Aqilah Bohani, Baher H. Nayef, Shahnorbanun Sahran, Omar Al Akash, Rizuana Iqbal Hussain, Fuad Ismail

**Affiliations:** ^1^Recognition Research Group, Center for Artificial Intelligence Technology, Faculty of Information Science and Technology, Universiti Kebangsaan Malaysia, 43600 Bangi, Malaysia; ^2^Department of Radiology, UKM Medical Center, Universiti Kebangsaan Malaysia, Cheras, 56000 Kuala Lumpur, Malaysia; ^3^Department of Radiotherapy and Oncology, UKM Medical Center, Universiti Kebangsaan Malaysia, Cheras, 56000 Kuala Lumpur, Malaysia

## Abstract

Brain magnetic resonance imaging (MRI) classification into normal and abnormal is a critical and challenging task. Owing to that, several medical imaging classification techniques have been devised in which Learning Vector Quantization (LVQ) is amongst the potential. The main goal of this paper is to enhance the performance of LVQ technique in order to gain higher accuracy detection for brain tumor in MRIs. The classical way of selecting the winner code vector in LVQ is to measure the distance between the input vector and the codebook vectors using Euclidean distance function. In order to improve the winner selection technique, round off function is employed along with the Euclidean distance function. Moreover, in competitive learning classifiers, the fitting model is highly dependent on the class distribution. Therefore this paper proposed a multiresampling technique for which better class distribution can be achieved. This multiresampling is executed by using random selection via preclassification. The test data sample used are the brain tumor magnetic resonance images collected from Universiti Kebangsaan Malaysia Medical Center and UCI benchmark data sets. Comparative studies showed that the proposed methods with promising results are LVQ1, Multipass LVQ, Hierarchical LVQ, Multilayer Perceptron, and Radial Basis Function.

## 1. Introduction

Magnetic Resonance Imaging (MRI) is heralded as tool of great importance in the context of human brain research as it provides detailed information about the soft tissue anatomy [1], which, in return, enhances the quality of the brain tumor diagnoses and treatment procedures. Owing to the overwhelming amount of data collected from the MRI, an automated image analysis tools are powerful for speeding up the analyzing process [2].

Numerous pattern recognition researches have been employed in MRI data analysis [3], but the process of automatic classification of brain magnetic resonance images is vital process for splitting normal and abnormal tissues with various brain diseases like cerebrovascular, Alzheimer, brain tumor, inflammatory diseases [4]. Brain tumor recognition and manual analysis from MR images are too time consuming and could introduce undesirable high intraobserver and interobserver variability. For that reason, researchers are pursuing to develop a new Computer Aided Diagnose (CAD) system that can better assist the medical experts in yielding highly accurate diagnosis with minimal risk. The quality of the segmented images depends on two requirements, first the removal of the noisy information from the source MR images and secondly the well conservation of the segmented images [5]. In this paper, LVQ classifier will be discussed to classify MR images into normal and abnormal.

Data preprocessing is the key step in data classification. High classification performance is significantly contingent upon the quality of the extracted features from the segmentation step. In addition, the data set class distribution affects the performance of the classifier. Multisampling or randomizing the data set by swapping the instances plays very important role in improving the classifier performance [6].

LVQ classifier assigns the input vectors to the code vectors by measuring the distance between them using Euclidean distance function. The code vector with minimum distance will be the winner and the input vector will be assigned to it. The distance differences between the input vector and some of the code vector may not be too significant and choosing the code vector with minimum distance only will push away unselected code vector for a certain number of iterations. These pushed away code vectors might be of imperative use and losing them will upset the classifier performance [7]. With that in mind, the objectives of this study are to enhance Learning Vector Quantization (LVQ) classifier, propose a framework for brain MRI classification based on LVQ, and evaluate the proposed multiresampling method with the state-of-the-art classifiers.

This paper is organized as follows: first, an intensive review to the past studies related to the brain MRI segmentation and LVQ classifier, then proposed methods section, the results and discussion section, and lastly, the conclusion.

## 2. Related Work

LVQ networks as originally proposed by Kohonen [9] are known good neural classifiers, which provide fast and accurate results for many applications. In contrast to the traditional neural network, LVQ is often deemed as a simple architecture, universal, and efficient classification due to its diminished complexity, learning time, and codebook representation [9–13]. Furthermore, LVQ is a widely [11] used approach in classification. It is applied in a variety of practical problems including medical image and data analysis [11], for example, in speech recognition [14] and control chart pattern recognition [15]. LVQ, a supervised learning approach, accepts predefined classes and labeled data. The goal of LVQ is to determine a set of prototypes that best represent each class. The approximate classification performed by LVQ is in accordance with its local boundaries [10]. In light of these advantages, LVQ can avert the use of all points in the training data set while retaining only a set of prototypes representing the best point for each class. Consequently, the number of stored vectors is definitely reduced and thereby increases the efficiency of LVQ complete performance. In vector quantization, it is assumed that there is a codebook which is defined by a set of *m* prototype vectors (*m* is chosen by the user and the initial prototype vectors are chosen arbitrarily).

LVQ family consists of LVQ1, LVQ2, and the improved versions LVQ2.1, LVQ3, OLVQ1, OLVQ3, Multipass LVQ, and HLVQ algorithms. LVQ algorithms are used for pattern classification, statistical pattern classification [16], and signal processing [17]. LVQ1 [18] used nearest neighbors concept where *m*1 and *m*2 are updated at the same time. One of them must belong to the correct class and the other to the wrong class. The input data vector *x* must be within a “window,” which is defined around the mid plane of *m*1 and *m*2. Then, LVQ3 [19] is introduced to overcome the reference vectors diverging drawback in LVQ2. A new parameter called epsilon (*ϵ*) has been introduced to tackle the case of both neighbors belonging to the same class and ensure that the reference vectors continue approximating the class distributions [19]. Then, an optimized LVQ3 (OLVQ3) is introduced which is slightly similar to LVQ3 regardless of the fact that each codebook vector has its own learning rate [20]. On the other hand, a combination of OLVQ1, LVQ1, and LVQ3 called Multipass LVQ (MLVQ) was also developed by U. Kumar and M. Kumar [21]. During the first pass [21], OLVQ1 is recommended for fast class distribution approximation, large learning rates (0.1–0.4), and short training times (30 or 50 times the number of the code vectors). Nevertheless, at second pass [21], LVQ1 and LVQ3 are recommended for slow fine-tuning of the class distribution and small learning rate values (less than 0.1 and 10 times the number of iteration in the first pass [21]). On the other hand, Hierarchy LVQ (HLVQ) which splits categories in feature space hierarchically in learning process has the adjacent feature spaces overlapping each other near the borders [22]. HLVQ controls both classification speed and accuracy because of the hierarchical architecture and the overlapping capability. Nowadays, some of researchers have tackled the problem of imbalanced biomedical data, that is, generating synthetic samples that presented a new oversampling method using codebooks obtained by Learning Vector Quantization [23].

According to [9], Self-Organizing Map neural network is used to analyze and visualize high dimensional data, that is, competitive learning network, described by the Finnish professor Teuvo Kohonen in 1981 and sometimes it is called Kohonen Map. It is a dimensionality reduction neural network. The SOM defines a mapping from high dimensional input data space onto a regular two-dimensional array of neurons. It is a competitive network where the goal is to transform an input data set of arbitrary dimensions to one- or two-dimensional topological maps. Another unsupervised learning is MLP. MLP has been principal because of the parallel implementation, the capacity for generalization, the fault tolerance, and the availability of efficient learning algorithms. However, a serious problem of MLP appears when applying it to image processing: the topological structure of the input patterns is ignored and inputs to the MLP are treated as one-dimensional vector [24]. The training phase with Radial Basis Function (RBF) starts in the hidden layer using unsupervised learning algorithm; then, it continues in the output layer using supervised learning algorithm, so, it is a hybrid supervised-unsupervised topology. RBF network will give good results for input data that are similar to its training data, but will give poor results for unfamiliar input data [25]. In addition, RBF neural network has some characteristics, such as simple training process, high adaptability, and strong distinguishing ability [26]. Another popular supervised learning classifier, namely, Random Forest (RF), builds the ensemble tree with the set of decision trees in which each individual tree is constructed by bootstrap samples via random selection from a collection of input data. On the other hand, these randomly samples are used for generating training data subsets for building individual trees [27].

MRI segmentation and classification have been the point of interest for myriads of pertinent researchers as a ground for testing and assessing the performance of different techniques. The study reviews subjects related to supervised and unsupervised classification with other ANN classifiers. LVQ classifier had been used in brain MRI segmentation and classification by many researchers. Neelam and Bikesh [28] discussed using LVQ for classifying brain tumors into malignant or benign by using shape and Haralicks texture features. On the other hand, Kashtiban et al. [29] study proposed Discrete Wavelet Transformation, wavelet packet, and feature selection using a multivariate statistical method are carried out to select the best wavelet coefficients as input feature vectors into the LVQ and Multiperceptron (MLP) classifiers. In Chalabi et al. [30], LVQ and Self-Organizing Map (SOM) were used to classify MR images and the higher resulting test images were gained using the combination of SOM and LVQ1. Qureshi et al. [31] study introduced LVQ with Discriminant Wavelet Packet Transform (DWPT) and back propagation for detecting specific types of meningiomas in images of neurosurgical resections. Bhaiya and Verma [32] proposed three ANNs, the LVQ, MLP, and RBF, with using Principal Component Analysis (PCA) to classify brain MRIs into normal and abnormal. Besides that, Random Forest (RF) and SVM were used in informative gene selection and cancer classification for selecting the important features [33].

Abdullah et al. [34] proposed Daubechies-4 (DAUB-4) and Haar with Support Vector Machine to segment and classify brain MRI. Cherifi et al. [35] proposed local, global, Otsu thresholding, and expectation maximization methods to segment specific area of the brain called the cerebrum. Hemanth et al. [36] proposed automatic classification of abnormal tumor images using the Adaptive Resonance Theory (ART2) neural networks with optimized number of features. Gopal and Karnan [37] introduced a new approach to segment brain MRI using fuzzy *c*-means (FCM) with GA and Particle Swarm Optimization (PSO). Shubhangi and Hiremath [38] proposed knowledge-based techniques and a multispectral analysis based on a Support Vector Machine (SVM) to classify MRI into normal and abnormal. Other than that, Sheejakumari and Sankara Gomathi introduced Particle Swarm Optimization and neural network (IPSONN) applied on tissues classification of normal and abnormal tissues on MR images [39]. Luts et al. [40] introduced a classification method based on color-coding the pixels according to a predefined histopathological class and the registered brain atlas for MRI segmentation. Sasikala et al. [41] discussed segmenting the glioblastoma-multiforme tumor from brain MR images and Freifeld et al. proposed Constrained Gaussian Mixture Model (CGMM) and curved evolution for detection and segmentation of Multiple Sclerosis (MS) lesions in Magnetic Resonance Imaging (MRI) of brain [42]. The researchers proposed special gray level dependence (SGLDM) and wavelet transform methods for extracting features, GA to find the optimal texture features, and three segmentation methods, optimal feature-based segmentation (the proposed method), region growing, and fuzzy *c*-means algorithm. The morphology operation is used to remove undesired pixel during segmentation process in overlapping cell images [43].

In conclusion, neither the LVQ contributors nor basic classifiers look into the issue of Euclidean distances and randomization to increase the overall classification performance. Merging these two ideas into MRI classification application can become a great potential in medical imaging problem solving.

## 3. Materials and Methods

The first part is MRI segmentation and feature extraction approach; the second part is classifying MRI into normal and abnormal using the extracted texture features from step one.

### 3.1. Image Acquisition

**Figure 1:**
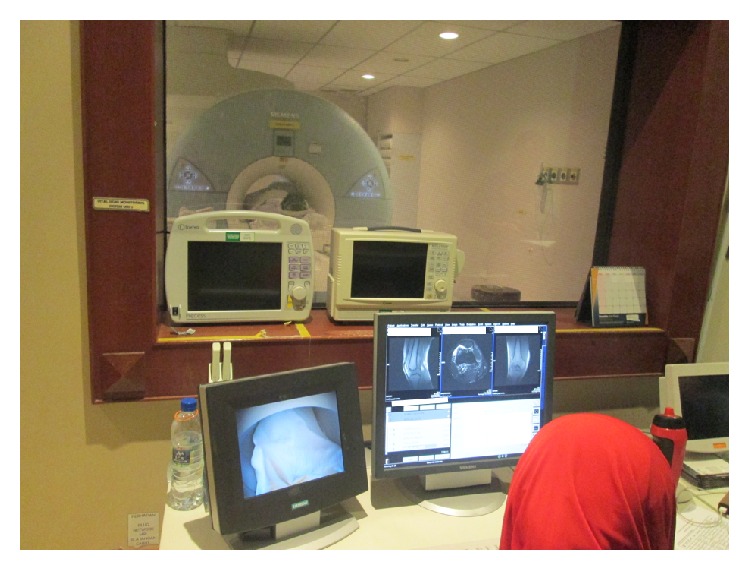
(a) MRI scanner at UKM Medical Center, Malaysia.

**Figure 2:**
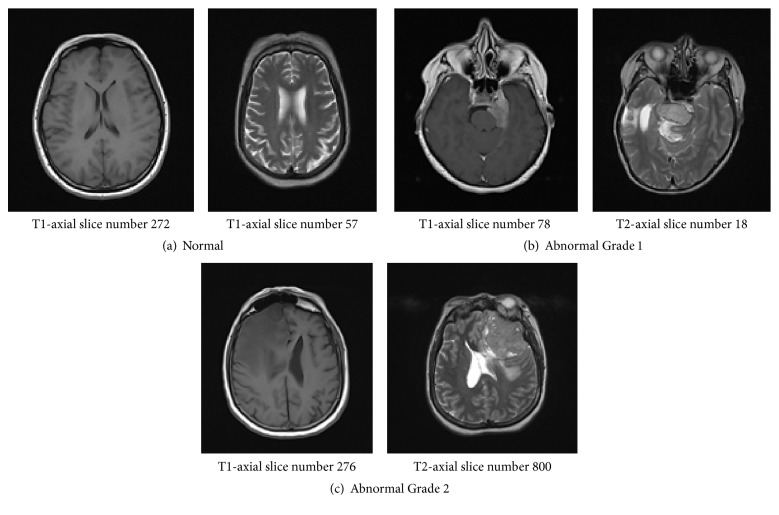
Examples of axial sequences in (a) normal and ((b) and (c)) abnormal brain MR images (i) T1-axial and (ii) T2-axial. Source: collected data from UKM Medical Center.

The images are captured by the radiologist from UKM Medical Center, Malaysia. Below are the sample of MRI Scannet Cutaway and MRI Scanner at Department of Radiologist, UKM Medical Center (Figures 1 and 2), respectively.

Referring to Figure 2 and Table 1, the collected data set consists of 272 abnormal and 233 normal MRIs from 9 patients. MRI scanner produces sagittal, coronal, and axial sequences. In this research, only T1 and T2 weighted axial sequence are used. For each patient, we selected 5 to 6 images for different slices because sometimes the tumor does not appear in the segmented image in one slice but it will be in the next slice; it depends on the intensity values of the pixels of that slice or cut.

### 3.2. Preprocessing

This step is very essential in image segmentation and classification as it has nonmarginal propensity to alter the accuracy of extracted features, which in turn affects the classifier performance. This step includes image enhancement, image filtering, and denosing. In this research, two filters have been used to enhance the images high pass filter as a frequency domain filter and median filter as a spatial domain filter.

The base fact of enhancing an image using high pass filtering is the changes in the gray levels combined with high frequency components of the Fourier transform of that image. This can be represented using the following formula.

Let $\hat{a}$ indicate the Fourier transform of the source image *a*.

If *a* indicates transfer function which reduces low frequencies and lets high frequencies pass, $\hat{h}$, then the filtered enhancement of *a* is the inverse Fourier transform of the product $\hat{a}$ and the enhanced image *g* is given by

(1) $$\begin{array}{c} g\left(\;x,y\;\right)=J^{-1}\left\{\;\hat{a}\left(\;u,v\;\right)\cdot \hat{h}\left(\;u,v\;\right)\;\right\}, \end{array}$$

where *J* ^−1^ indicates the inverse Fourier transform.

The high pass transfer function formula is solely the complement of the low pass transfer function. The transfer function of the ideal high pass filter is given by

(2) $$\begin{array}{c} \hat{h}\left(\;u,v\;\right)=\left\{\begin{array}{cc} 0 & \text{if }{\hat{d}}^{}\left(u,v\right)\leq d,\\ 1 & \text{if }{\hat{d}}^{}\left(u,v\right)>d, \end{array}\right. \end{array}$$

where *d* represents the cutoff frequency which is a defined nonnegative quantity and ${\hat{d}}^{}\left(u,v\right)={\left(u^{2}+v^{2}\right)}^{1/2}$. The transfer function of the Butterworth high pass filter of order *k* is given by

(3) $$\begin{array}{c} \hat{h}\left(\;u,v\;\right)=\left\{\begin{array}{cc} \frac{1}{1+c{\left[d/{\hat{d}}^{}\left(u,v\right)\right]}^{2k}} & \text{if }\left(u,v\right)\neq \left(0,0\right),\\ 0 & \text{if }\left(u,v\right)=\left(0,0\right), \end{array}\right. \end{array}$$

where *c* is a scaling constant and the default values for *c* are 1 and $\sqrt{2}-1$.

The transfer function of the exponential high pass filter is given by

(4) $$\begin{array}{c} \hat{h}\left(\;u,v\;\right)=\left\{\begin{array}{cc} e^{\left[-a{\left(\left(d/{\hat{d}}^{}\right)\left(u,v\right)\right)}^{k}\right]} & \text{if }\left(u,v\right)\neq \left(0,0\right),\\ 0 & \text{if }\left(u,v\right)=\left(0,0\right). \end{array}\right. \end{array}$$

**Figure 3:**
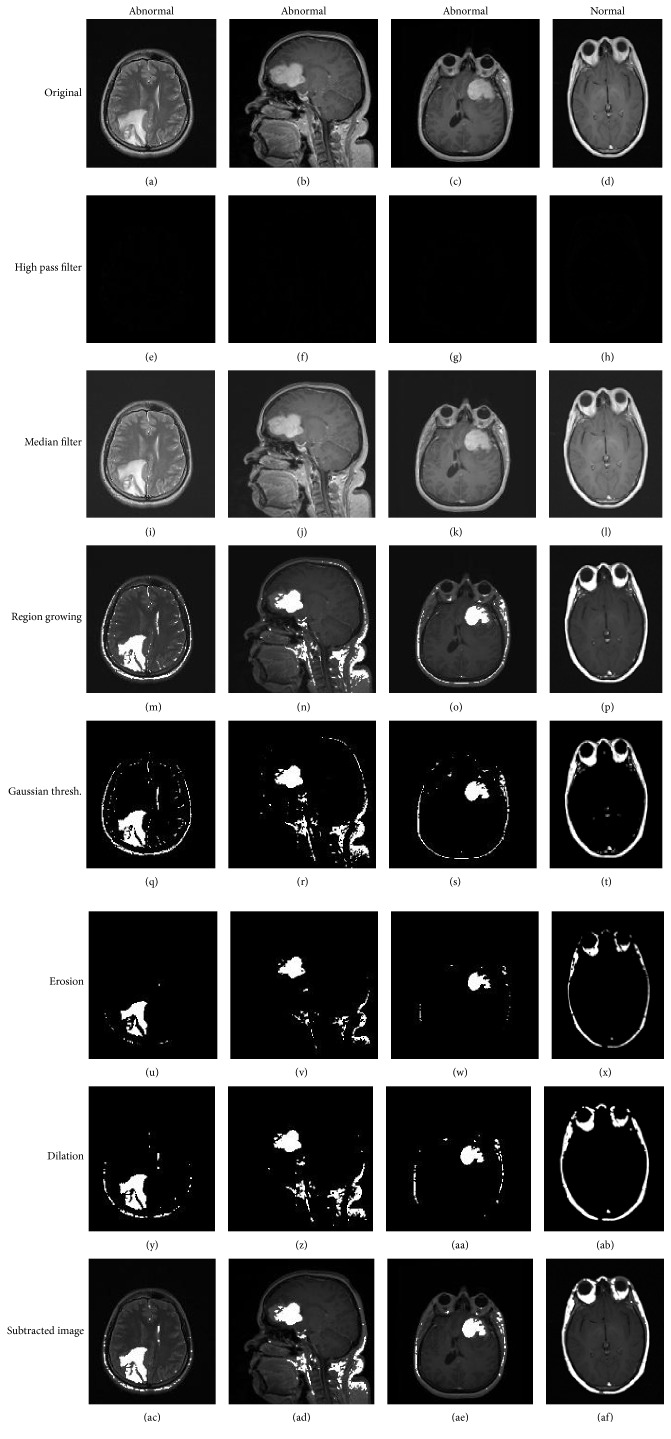
From the left to the right: ((a)–(d)) original images, ((e)–(h)) the output images with 3 × 3 high pass filter, ((i)–(l)) the output images after applying median filter with 3 × 3 kernel, ((m)–(p)) samples of region growing segmentation with the seed point value = 80 and threshold = 90, ((q)–(t)) samples of brain images after applying Gaussian search thresholding method, ((u)–(ab)) opened images with 6 × 6 mask of structure elements, and ((ac)–(af)) the final subtracted images. The last column represents normal image.

The default values for *a* are 1 and $ln\sqrt{2}$ suggested by Wilson and Ritter [44]. In this research, high pass filter is applied to sharpen edges in the image. Samples from brain MRI images are shown in Figure 3(a).

A nonlinear spatial smoothing filter orders the pixel values around the neighborhood and takes the median value as a result [45]. Median filter equation (5) is as follows:

(5) $$\begin{array}{c} {\hat{f}}^{}\left(\;x,y\;\right)={\text{median}}_{\left(s,t\right)\in S_{xy}}\left\{\;g\left(\;s,t\;\right)\;\right\}, \end{array}$$

where ${\hat{f}}^{}\left(x,y\right)$ represents the value of the restored image *f* at any point (*x*, *y*), *S*_*xy*_ dictates as a set of coordinates in a rectangular subimage window of size *m* × *n* centered at point (*x*, *y*), and *g*(*s*, *t*) represents the source corrupted image [46].

By using additive image function, the input image to the median filter is a combination of the source image and high pass filter output image and *c* is a constant for enhancing the image brightness as shown in (6). The result of applying median filter is shown in Figures 3(i)–3(l):

(6) $$\begin{array}{c} g\left(\;s,t\;\right)=\hat{a}\left(\;u,v\;\right)+g\left(\;x,y\;\right)+c, \end{array}$$

where *g*(*s*, *t*) is the input image to the median filter, $\hat{a}\left(u,v\right)$ is the source image, *g*(*x*, *y*) is the output of the high pass filter, and *c* is a constant set to 25.

Image segmentation is implemented by using two algorithms: region growing and Gaussian search algorithms. Region growing based algorithm is oriented towards continuity. The image is divided into subregions depending on some rules on account of the fact that all the pixels should have the same gray level. Region based techniques depend on the intensity values patterns within a cluster of neighboring pixels. Each region is a cluster and the main goal of the segmentation algorithm is to gather regions according to their anatomical or functional roles [47].

This method starts with a pixel and continues adding the pixels based on similarity of the region. When the growth of a region stops, another seed pixel which does not belong to any other region is selected, and again the process is started. The whole process is repeated until all pixels belong to another region. It marks the brightest pixel or the tumor region in the image by setting the seed point value to 80 and the threshold to 90. Thus, only the points with gray scale values less than 90 and above the seed point value 80 will be chosen as shown in Figures 3(m)–3(p).

A normalized multimodal histogram *p*(*x*) of an image (*x* represent the gray levels) can be suited with the sum of the probability density functions (pdf) to obtain the optimal thresholds for segmenting an image. The Gaussian pdf's form as follows:

(7) $$\begin{array}{c} p\left(\;x\;\right)=\overset{d}{\underset{i=1}{\sum }}\frac{P_{i}}{\sqrt{2\pi \sigma_{i}}}\cdot \exp\left[\;-\frac{\left(x-\mu_{i}\right)}{2\sigma_{i}^{2}}\;\right], \end{array}$$

where *P*_*i*_ is the a priori probability and ∑_*i*=1_^*d*^ *P*_*i*_, *d* is the levels of thresholding, *μ*_*i*_ is the mean, and *σ*_*i*_^2^ is the variance of mode *i*. In order to find the optima threshold, the maximum likelihood or mean-squared error approach is used for fitting pdf model to the histogram data [48]. Figures 3(q)–3(t) shows samples of applying Gaussian search thresholding method outputs.

Erosion and dilation are combined for forming opening and closing operations subsequently. They act as powerful operators. Opening moves apart the objects that are too close together, isolates objects that are touching, and enlarges holes inside objects. The definition of a morphological opening of an image is erosion followed by dilation using the same structuring element for both operations. Opening removes small islands and thin filaments of object pixels [49]. Figures 3(u)–3(ab) show the resultant images after applying the opening operation. The output of the opening operation image is subtracted from the source image, namely, image subtraction. Image subtraction is a common tool for the analysis of change in pairs of images, used in a wide range of circumstances [50]. The subtraction formula is

(8) $$\begin{array}{c} Q\left(\;i,j\;\right)=P_{2}\left(\;i,j\;\right)-P_{1}\left(\;i,j\;\right), \end{array}$$

where (*i*, *j*) are as pixel coordinates, *P*_1_ and *P*_2_ are the pixels of the source image and the opened image from Figure 3, and *Q*(*i*, *j*) is the resulting subtracted image. Figures 3(ac)–3(af) display the final subtracted images.

### 3.3. Postprocessing

After performing preprocessing, a texture statistical approach using Gray Level Cooccurrence Matrix (GLCM) technique is applied to indicate features representations. The intensity histograms of an image or region are usually used to describe texture. The information obtained from histograms calculation represents the distribution of the intensities and nothing about the position relation between the pixels in that texture. Cooccurrence matrix is a good statistical approach which provides valuable information about the position relation of the neighboring pixels in an image [51].

Given an image *I*, of size *N* × *N*, the cooccurrence matrix *P* can be defined as

**Figure 4:**
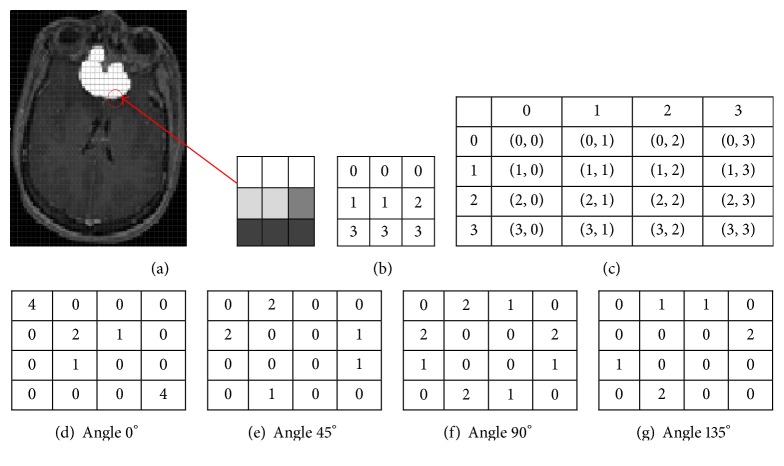
(a) A gray level image (final image) for generating the cooccurrence matrix from a 3 × 3 matrix kernel and cooccurrence matrix ((b)–(g)) defined by GLCM.

(9) Pi,j=∑x=1N ∑y=1N1,if  Ix,y=i,  Ix+Δx,y+Δy=j,0,otherwise,

where the offset (Δ*x*, Δ*y*) defines the distance between the pixel of interest and the surrounding neighbors. Because of this offset, the cooccurrence matrix is sensitive to rotation. Therefore, a set of offset sweeping through 180 degrees at the same parameter Δ to achieve a degree of rotational invariance (i.e., $\left[\begin{array}{cc} 0 & \Delta \end{array}\right]$ for 0°: *P* horizontal, $\left[\begin{array}{cc} -\Delta & \Delta \end{array}\right]$ for 45°: *P* right diagonal, $\left[\begin{array}{cc} -\Delta & 0 \end{array}\right]$ for 90°: *P* vertical, and $\left[\begin{array}{cc} -\Delta & -\Delta \end{array}\right]$ for 135°: *P* left diagonal) are tested to form the co-occurrence matrix as explained in Figure 4.

As it is observable, Figure 4(b) represents a 3 × 3 image with four gray tones from 0 to 3 which is obtained from Figures 4(a) and 4(c) illustrating gray tone spatial-dependence matrix which focuses on the connectivity of each cell with different angles. In Figure 4, (d) is the cooccurrence matrix with angle 0, (e) with angle 45°, (f) with angle 90°, and (g) with angle 135°. For example, the element in the (0,0) position of Figure 4(c) is the connectivity of these two elements with different angles, Figure 4(b) with angle 0°; two elements are connected with angle 0°, so the total number of times at which two gray tones of value (0,0) occurred horizontally is 4.

After producing the cooccurrence matrix, feature can be extracted from the source MR image combined with the output image of the opening operations. The extracted features are listed as [52].

For increasing the discriminative power, the Principal Component Analysis (PCA) has been used. PCA is preferable since it effectively reduces the dimensionality of the data and computational cost of analyzing new data [53]. By applying dimensionality reduction using (PCA), only four features are used in the classification stage.

Given a set of GLCM training images, *I*_*i*_(*x*, *y*), *x* = 0,1,…, *n* − 1, *y* = 0,1,…, *m* − 1, and *i* = 0,1,…, *N* − 1, where *I*_*i*_(*x*, *y*) is of size *N* × *M*. In the training images, the active portions have been manually labeled for extracting the parameters of the shape and appearance models. In a 2D image, *n* landmark points can be represented as a 2*n* shape vector, *X*, where *X* = (*x*_1_,…,*x*_*n*_, *y*_1_,…,*y*_*n*_)^*T*^. The set of shape vectors has been normalized to a common reference frame; hence, *X* can be represented by *x* by applying PCA:

(10) $$\begin{array}{c} S=\overset{-}{S}+F_{s}n_{s}, \end{array}$$

where *S* represents the synthesized shape in the normalized frame, $\overset{-}{S}$ illustrates the mean shape in the normalized frame, *F*_*s*_ depicts the matrix of eigenvectors, extracted from the training shape, and *n*_*s*_ is a set of shape parameters. Soon after acquiring a shape model, every single training image has been distorted, where its control factors could match the mean shape.

### 3.4. The Proposed LVQ Classifier

**Figure 5:**
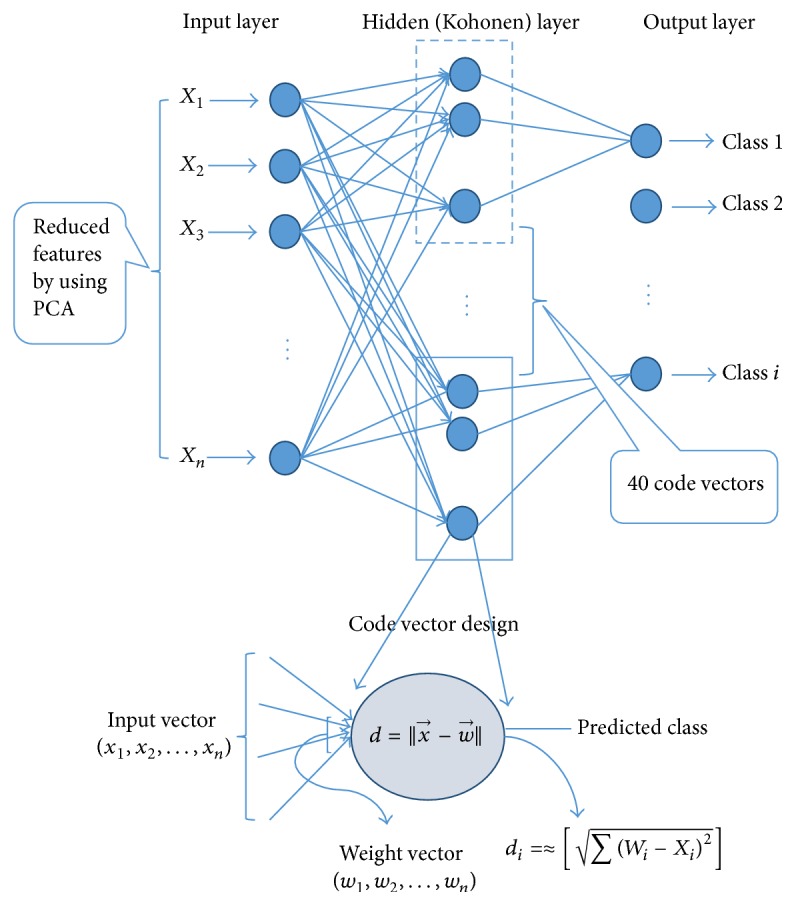
The proposed LVQ classifier structure with the modified distance function.

**Figure 6:**
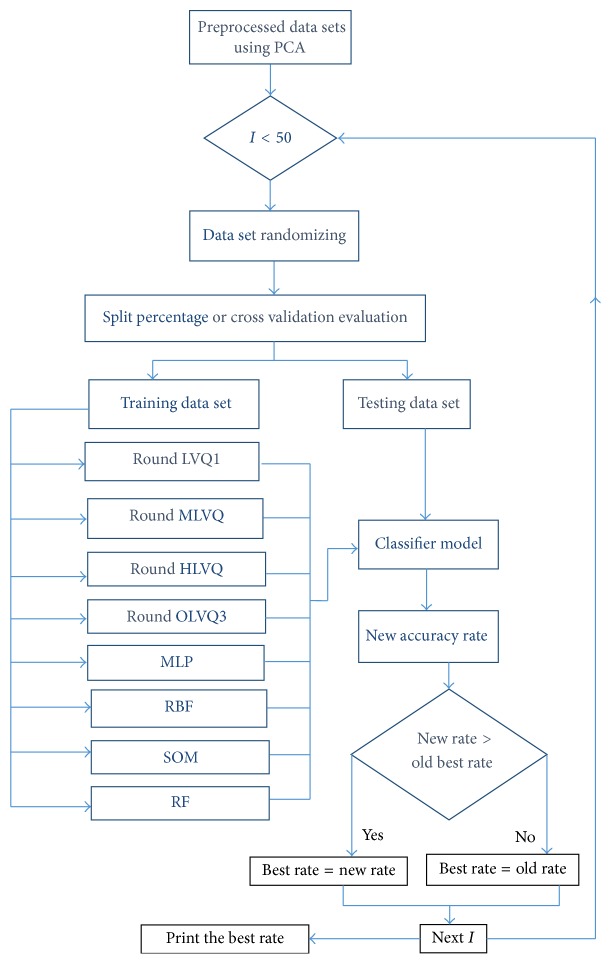
The proposed classification based on multirandomization flowchart using Round LVQ series and MLP, RBF, SOM, and RF classifiers.

In LVQ classifier, the input vector is assigned to a certain code vector depending on the minimum distance between them measured using Euclidean distance equation. Some of these code vectors are pushed away permanently if they did not assign any input vector for a certain number of iterations. The distance differences between the input vector and the code vectors of the competitive layer could be very small but only the code vector with minimum distance chosen and the other code vectors are pushed away. These pushed away codes vectors could be important to classify data and excluding them may affect the classifier performance. Therefore, to give these code vectors a chance to be chosen and stay longer in the competition, the round-off function is used with Euclidean distance equation (11) in Figure 5 and the modified LVQ structure shown in Figure 6. In addition, the round-off function is applied to minimize the calculation dimensionality.

The proposed round distance *d*_*i*_ between the weight vectors *W*_*i*_ of neuron *i* and the input vector *X* is given by

(11) $$\begin{array}{c} d_{i}=\approx \left[\;\sqrt{\underset{}{\sum }{\left(W_{i}-X_{i}\right)}^{2}}\;\right], \end{array}$$

where *W*_*i*_ and *X*_*i*_ are the *i*th components of *W* and *X*, respectively.

Algorithm 1 is provided for preparing codebook vectors using the LVQ. Codebook vectors initialized to small floating-point values. The Best Matching Unit (BMU) is the codebook vector, which has the minimum distance to an input vector. The modified Euclidean distance function equation (11) is used to measure the distance between the input vector and the codebook vector.

The class distribution plays a major role in building good models in the training phase. If the training data set does not include all the different classes, that, in turn, will pose a significant effect on the classification performance in the testing phase. Therefore, multiresampling method is proposed to ensure a balanced class distribution. In our proposed method, we randomize the data set for 50 times and that leads to generate 50 forms of the source data set by swapping the instances in each time. After each randomization, a training model is built and evaluated. In the proposed method, only the best model is considered. Below is shown the proposed LVQ classification as in Algorithm 2 and Figure 5 explains the classification phase with multirandomizing data sets steps in detail.

After applying segmentation methods on images, different series of neural networks (NN) are also used to classify the segmented images for comparison. These ANN are LVQ classifiers family, which consists of LVQ1, Multipass LVQ, Hierarchal LVQ, Multiperceptron (MLP), Self-Organizing Map (SOM), Radial Basis Function (RBF) Networks, and Random Forest (RF) [27] as shown in Figure 6.

## 4. Experiments and Results

In this study, three types of experiments were conducted. The first experiment (I) is applied to evaluate the performance of the standard LVQ series and conducted to test the effect of the proposed methods together and individually on the performance of LVQ series. The second experiment (II) performed to compare the performance of LVQ series with Multiperceptron (MLP), Self-Organizing Map (SOM), Radial Bases Function (RBF), and RF classifiers.

### 4.1. Experiment I

In the previous section, the performance of all LVQ series was evaluated without rounding off the distance function and multirandomizing the data sets. This section discusses the effect of rounding off the distance function with multirandomizing the data sets on LVQ series. The objective of this experiment is to investigate the performance of LVQ series with the effect of proposed work in addition to a comparison of the LVQ classifiers performances before and after running the proposed work. The initial parameters of the LVQ classifiers used in this experiment are 0.3, 40, 2000, and learning decay for the learning rate, number of codebook vectors, number of iterations, and learning function, respectively. The similar brain tumor, segmented challenge, and segment-test data sets used in the previous experiment are used to perform this experiment.

LVQ classifier structure consists of three layers: input layer, competitive layer, and output layers. The model accuracy is highly depending on the initialization of the model as well as the learning parameters used (learning rate, training iterations, and number of codebook vectors). Therefore, selecting the best number of iterations and learning rate is an important issue to improve the classifier accuracy. In addition, multirandomizing the data sets is very helpful to find the best data set form producing the best model accuracy rate.

**Figure 7:**
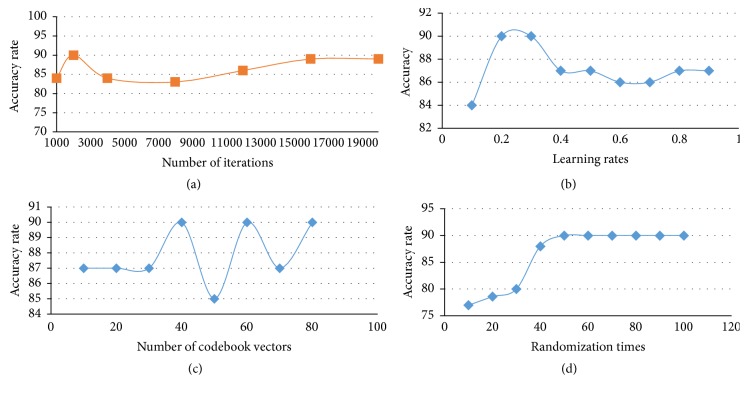
(a) Graph of accuracy rates versus number of iterations for the proposed LVQ1. (b) The proposed LVQ1 accuracy rate versus the learning rate. (c) The proposed LVQ1 accuracy rate versus number of codebook vectors. (d) Number of randomization times versus accuracy rate for the proposed LVQ1.

Since LVQ1 is the first version, it has been selected to find the suitable number of training iteration learning rate, and number of codebook vectors, by using the main brain images data set. Split percentage evaluation method is used to split the data set into training (70%) and testing (30%) data sets. By running different experiments, it is clear that the best number of iterations is 2000. The proposed classifier accuracy achieved is 90% with Mean Square Error (MSE) equal to 0.3113 as shown in Figure 7(a).

Choosing the learning rate is very helpful for faster convergence. The decreasing learning rate allows the network to converge to a state in which the weight vectors are stable [54]. In this research, the learning rate has been tested for the values from 0.1 to 0.9 in order to find the appropriate learning rate using the proposed LVQ1 classifier with brain images data set divided into 70% for training and 30% for testing and for 2000 iterations. From Figure 7(b), the acceptable learning rate (LR) is either 0.2 or 0.3 in which the model accuracy rate achieved 90% with these two learning rates.

Adequate initialization of the codebook vectors is highly important issue with the LVQ algorithm. The optimal numbers of prototypes per class are not easy to derive because of the complexities of class-conditional densities and variability in class distributions. The simplest way to initialize prototypes is to select them by random sampling from the available training points [9]. Thus, in this research, selecting the best number of codebook vectors is based on different runs using brain images data set with 70% split percentage, 0.3 learning rate, and 2000 training iterations. The results of these runs are explained in Figure 7(c). The best number of codebook vectors to obtain 90% accuracy rate is either 40, 60, or 80.

Multirandomizing data sets before classification aims to find the suitable form of the data set with the class distribution. This will improve the classifier model performance. In this research, a number of randomization times from 10 to 100 are applied on brain data using 0.3 for the learning rate and 2000 iterations. From Figure 7(d), it is obvious that 50 times is the acceptable number of randomization times in which the accuracy rate reached 90% and stabilized for the subsequent runs with 70% split percentage.

**Figure 8:**
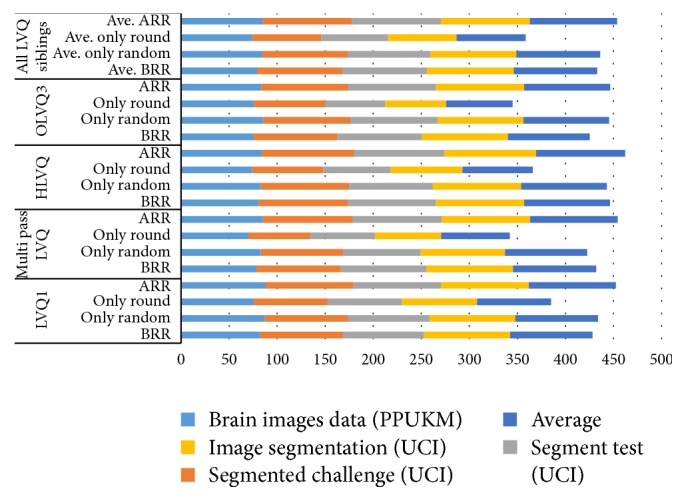
The accuracy rate versus all LVQ series before and after applying the proposed method on brain, segmented challenge, and segment-test data sets with 70% split percentage (refer to the Appendix).

The average accuracy rates of all LVQ series with brain, segmented challenge, segment test, and image segmentation data sets obtained from the proposed work are shown in Figure 8 and Table 2. The best accuracy rate is 96% obtained from HLVQ with segmented challenge data sets and the lowest accuracy rate is 91.36% obtained by LVQ1 with segmented test data sets. LVQ1 obtains the least standard deviation (STD) value (1.56) followed by multipass OLVQ3 (3.90) and then Multipass LVQ (4.02) and the highest STD belongs to HLVQ (5.52).

**Figure 9:**
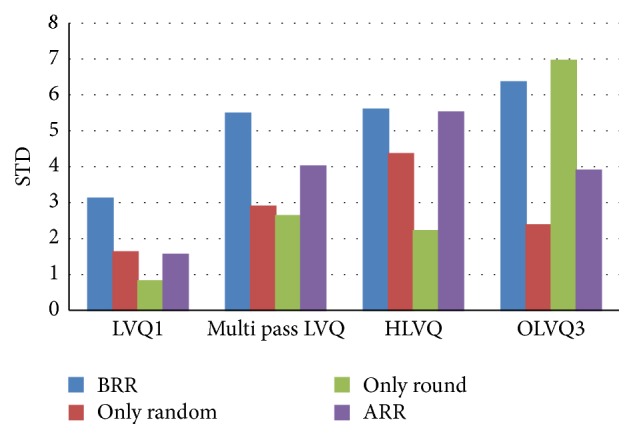
The improvement of Std values for LVQ siblings classifiers with 70% split percentage.

Table 2 and Figure 8 showed the average accuracy rate of LVQ series with and without the proposed work. From the table, it is noticeable that the STD value of LVQ1, MLVQ, HLVQ, and OLVQ3 with the proposed work is significantly less than that without the proposed work. This indicates that the proposed work affects the classifiers stability in a positive way. In general, all LVQ series show better stability with the proposed work as shown in Figure 9 with 70% split percentage. On the other hand, the accuracy mean values of all data sets which are brain, segmented challenge, segmented test, and image segmentation data set have shown a clear improvement with the proposed work compared to those without it.

Figure 8 explains the performance of the LVQ classifiers with rounding off the distances function values and multirandomizing data sets separately. Rounding off the distance function values affects positively the stability of LVQ1, MLVQ, and HLVQ classifiers. The standard deviation values after applying the proposed work are less than those before applying it. Unlike OLVQ3 classifier has shown unstable performance and higher standard deviation values after applying rounding off method. In addition, the stability of the all LVQ classifiers with the proposed work yields significant improvement compared to that without applying the proposed work as shown in Figure 9.

According to the multirandomization method, the accuracy rates for LVQs with the brain, segmented challenge, segment test, and image segmentation data set are clearly improved beside the improvement of the standard deviation values.

### 4.2. Experiment II

In this section, multirandomization data sets method is applied on MLP, RBF, SOM, and RF classifiers in addition to four classifiers from the LVQ series: LVQ1, Multipass LVQ, Hierarchy LVQ, and OLVQ3. A full discussion and comparison between the performance of these classifiers before and after applying the proposed work are presented.

In this experiment, four data sets with different dimensionality, sizes, and number of classes are used which are brain data set, segmented challenge data set, segment-test data set, and image segmentation data set. For LVQ classifiers, the learning rate used in this experiment is 0.3, the number of iterations is 2000, and the total number of code vectors is set to 40. The configuration of MLP, RBF, SOM, and RF classifiers is shown in Table 3.

**Figure 10:**
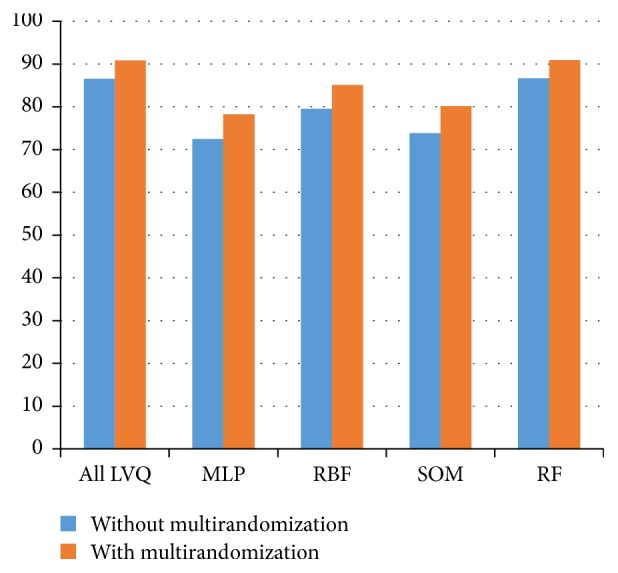
Average accuracy comparison using with and without proposed multirandomization for all classifiers including LVQs, MLP, RBF, SOM, and RF classifiers with 70% split percentage.

**Figure 11:**
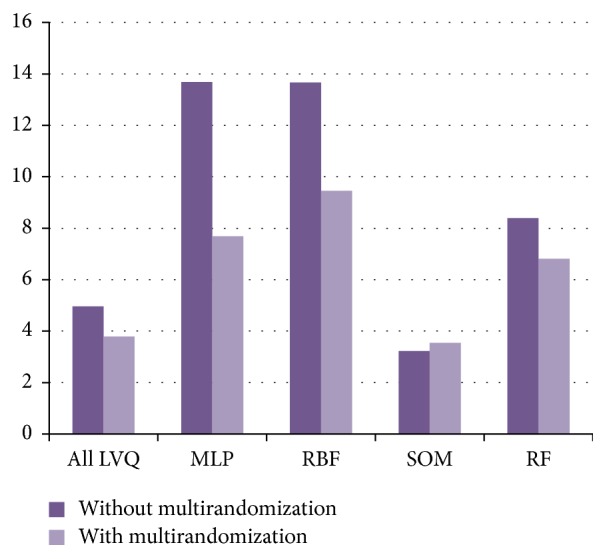
Standard deviation values for LVQ, MLP, RBF, SOM, and RF classifiers with and without the proposed multirandomization.

The average accuracy rates of LVQs and RF classifiers exhibited a slight improvement. For MLP, RBF, and SOM classifier, it is noticeable that there is a clear improvement in the accuracy rates. All classifiers including LVQs, MLP, RBF, SOM, and RF use 70% split percentage in evaluation method. Based on LVQs and RF performances, both classifiers obtained mostly the same values in terms of accuracy. However, standard deviation of RF is larger than LVQs. All the results are shown in Table 4 and Figure 10. On the other hand, Figure 11 depicts a conclusion that all classifiers are performing better accuracy and more stable and consistent results after performing our proposed multirandomization method.

The performance of the LVQs algorithm has been improved from average of 84.5% to 85%–93% for all of data set.

## 5. Discussion

Brain MRI images which were collected from UKMMC are the main data set of this study. The image processing steps started from removal of the noise, followed by edge sharpening; then, segmentation thresholding before the morphological operation is performed. The final images are extracted using GLCM; then, dimensionality of input features is deducted and distinguishing capability is increased by using Principal Component Analysis (PCA). There are a few LVQ siblings, namely, LVQ1, Multipass LVQ, HLVQ, and OLVQ3, which are executed for training the nearest-neighbor that applied in such pattern recognition, diagnostics, control system, and monitoring task [55].

The some of the disadvantages of LVQ classifier are as follows:

(1) There are slow convergence and possibility of being trapped at a locally minimum value [9].
(2) The corrections of **x**_**i**_ (input vector) have a larger magnitude than that on **w**_**i**_ (codebook vector), thus resulting in monotonically decreasing distances **d** = ‖**x**_**i**_ − **w**_**i**_‖ in which the minimum distance is called Best Matching Unit (BMU). Although this effect is not as important with high dimensionality, it would be an improvement if the corrections on **x**_**i**_ and **w**_**i**_ had the same magnitude. This can be affected, for example, by normalization [9].
(3) The sensitivity of the number and location for initial codebook vectors is first determined without taking their classification into account, and then calibrating the codebook vectors by input vectors with known classification [9] is also important step to increase the performance.

Prior to the above statements and findings, HLVQ outperforms other LVQ siblings. HLVQ controls both classification speed and accuracy because of the hierarchical architecture and the overlapping technique [22]. In general, LVQ involved the input vectors and the number of codebook vectors yielded from Euclidean distance function. Some neurons in LVQ may be too frequent while others are always inoperative. Hence, minimize the dependency of training example as the initial weights. Regarding training sample which consists of small number of classes, the problems are overcome by using multirandomization method that has successfully shown better performance of distribution of training examples. To enlarge the possibility of the neurons as the winner,* rounding of function* is applied to the Euclidean distance equation.

LVQ1 achieved higher accuracy rate compared to three other classifiers, namely, MLP, RBF, and SOM. Though RF classifier showed the highest accuracy, its standard deviation dictates the highest (about 5–9 std) with and without multirandomization methods. The impact factors of LVQ performances are learning rate, number of codebook vectors in competitive layer, and the initial weight. On the other hand, Euclidean distance is used for providing distance between input vector and codebook vector. Here, the minimum distance of these both neurons will be the winner and the others will be removed regardless of information importance existence. LVQ has two major drawbacks that many researchers [56] deduce them to be slow convergence and instable behavior. The convergence problem has been solved using Genetic Algorithm [57] which increased the performance of classification rate prior to power quality disturbances.

## 6. Conclusion

This chapter displayed all the results of the experiments done on LVQ series and MLP, SOM, RBF, and RF classifiers. The first experiment conducted to evaluate the performance of LVQ series with the proposed work. The results of comparing the performance of all LVQ series with and without the proposed work showed that HLVQ classifier performance is more stable than the other LVQ series. It is justifiable as the standard deviation of proposed work is outperformed compared to classic LVQ. In addition, the performance of proposed work (with the combination of multirandomization and round-off) applied in all LVQ series is increased between 2% and 8% for all of data sets. Another experiment was performed as part of the first experiment to test the performance of LVQ classifiers with rounding off the distance function values versus multirandomizing the data set. The results showed that accuracy means with rounding off method are less than multirandomization method but all LVQ classifiers with round-off showed better stability than those with multirandomization method. The stability of LVQ based on standard deviation with multirandomization method also warrants an emphasis, whereby their rounded off values are about 2.82 and 1.9, respectively. Likewise, experimental results have revealed that the injection of combined multirandomization and rounded off Euclidean function on LVQ classifier is highly effective for brain tumor classification.

On the other hand, the second experiment is performed to test the proposed work with MLP, SOM, RBF, and RF using brain images, segmented challenge, segment test, and images segmentation data sets. Hence, it is justified to conclude that the efficiency of the proposed work using multirandomization method during prelearning process attests to the improved performance of LVQ classifiers in supervised (MLP, RBF, and RF) and unsupervised learning methods (SOM) in all data sets.

The experimental result demonstrated that the multirandomization method during the preparation of learning data set throughout the preprocessing step by means of MLP, RBF, SOM, and RF is highly effective for brain tumor classification which encompasses segmented challenge, segment test, and images segmentation provided. This is evidenced by the expected result which increases between 4% and 6% in accuracy of classification. Both performances of LVQ classifiers and RF with multirandomization only are acceptable. The results showed that the classification rate of RF outperformed the other classifiers. Therefore, we are looking forward to venture in the improvements of RF methods via reducing standard deviation score to yield better and more consistent performance.

## Figures and Tables

**Figure 12:**
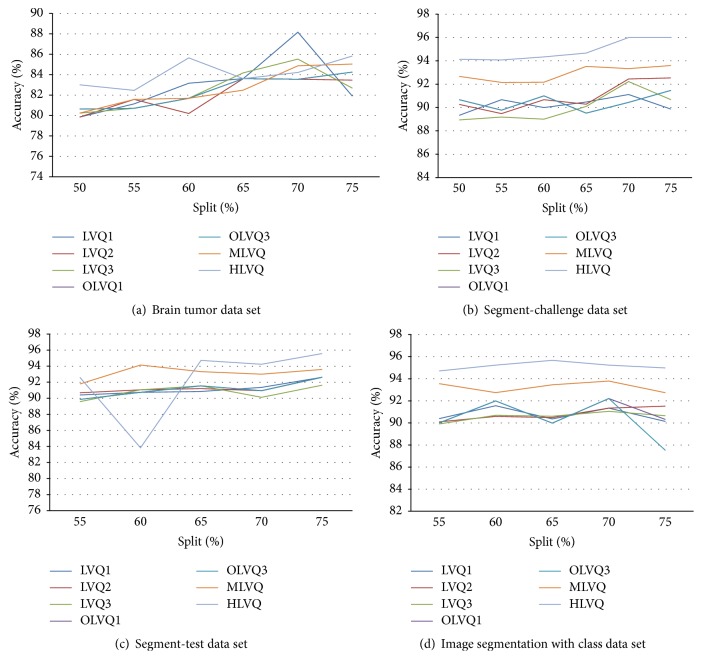
The graph shows split percentage evaluation method that selected training (70%) and testing (30%) as suitable split percentage to split data for each data set.

**Algorithm 1:**
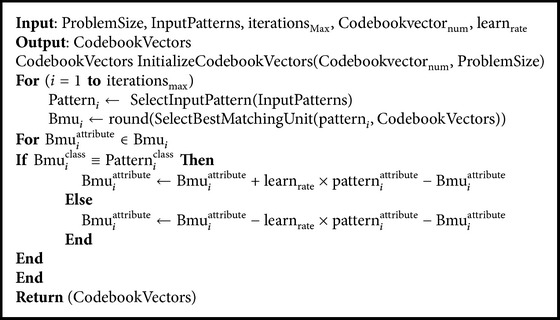
LVQ1 classifier [8].

**Algorithm 2:**
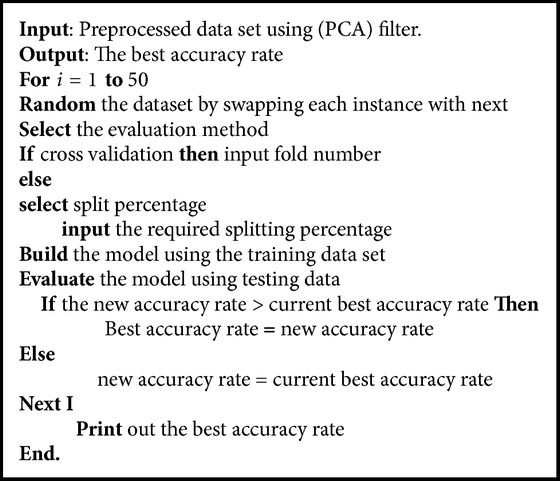
Classification based on multiresampling method.

**(a):** 

Dataset name	Number of instances	Spatial resolution	Number of features	Number of classes	Source
Brain tumor images	505	768 × 768	21	2 (Normal, abnormal)	UKMMC
Class type		T1 Sub total		T2 Subtotal	
Normal		158		75	
Abnormal		187		85	

**(b):** 

Abnormal subclass type	Cancerous type	Number of patient data	Range of tumor size based on medical expert report
Abnormal (Grade 1)	CP meningioma	1	1.5 cm (AP) × 2 cm (W) × 1.9 cm (CC)
	Suprasellar mass	1	5.8 cm (AP) × 7.2 cm (W) × 6.2 cm (CC)
	Schwannoma with mild hydrocephalus	1	2.7 cm (AP) × 4.1 cm (W) × 4.8 cm (CC)

Abnormal (Grade 2)	Astrocytoma	1	4.4 cm (AP) × 2.7 cm (W) × 4.8 cm (CC)
	Hemangiopericytoma	1	1.2 cm (AP) × 1.3 cm (W) × 1 cm (CC)
	Atypical meningioma	1	2.6 cm (AP) × 1.8 cm (W) × 2.8 cm (CC)

**Table 2:** Accuracy rates for LVQ classifiers before and after applying the proposed work and the results of round-off and multirandomization methods individually.

Classifier	Datasets	Brain images data (PPUKM)	Segmented challenge (UCI)	Segment test (UCI)	Image segmentation (UCI)	Average	STD
LVQ1	BRR	82.24	86.22	84.36	89.61	85.61	3.12
	Only random	87	87	85	89	86	1.63
	Only round	76	77	77	78	77	0.82
	ARR	**88.16**	**91.11**	**91.36**	**91.34**	**90.49**	**1.56**

Multi pass LVQ	BRR	78.29	87.78	89.3	90.19	86.39	5.49
	Only random	83	85.5	81	87.7	85.4	2.9
	Only round	70	64	68	69	71	2.63
	ARR	**84.87**	**93.33**	**93**	**92.21**	**90.85**	**4.02**

HLVQ	BRR	80.92	92.89	91.36	91.92	89.27	5.6
	Only random	83	92	87	92	89	4.36
	Only round	74	74	70	75	73	2.22
	ARR	**84.21**	**96**	**94.24**	**95.24**	**92.42**	**5.52**

OLVQ3	BRR	75.66	86.89	88.07	89.61	85.06	6.36
	Only random	85.75	90.45	91.1	89.1	89	2.38
	Only round	76	73.9	62.5	63.5	69	6.96
	ARR	**83.55**	**90.44**	**90.95**	**92.21**	**89.29**	**3.9**

ALL LVQ siblings	Ave. BRR	79.2775	88.445	88.2725	90.3325	86.58	4.96
	Ave. only random	84.6875	88.7375	86.025	89.45	87.35	2.8175
	Ave. only round	74	72	69.4	71.4	71.7	1.92
	Ave. ARR	**85.1975**	**92.72**	**92.3875**	**92.75**	**90.76**	**3.71**

(i) BRR means before rounding the distance function results and multirandomizing data sets.

(ii) ARR means after rounding the distance function results and multirandomizing data sets.

**Table 3:** MLP, SOM, and RBF configuration.

MLP	RBF	SOM	Random Forest
Decay = true Hidden layers = 5 LR = 0.3 Momentum = 0.2 Training time = 2000 Validation threshold = 20	Seed = 1. The random seed to pass on to *K*-means. maxIts = −1. Maximum number of iterations for the logistic regression to perform. Only applied to discrete class problems. minStdDev = 0.1. Sets the minimum standard deviation for the clusters. numClusters = 2. The number of clusters for *K*-means to generate. Ridge = 1.0*E* − 8. Set the ridge value for the logistic or linear regression.	Learning function = linear decay (tangen) Learning rate = 0.3 Map height = 6 Map width = 8 Neighborhood function = Gaussian Neighborhood size = 8 Seed = 1 Supervised = false Number of iterations = 2000	Debug = true Maximum depth = 0 Number of features = 1 Seed = 1

**Table 4:** Average accuracy rates with and without multirandomization for all LVQ sibling and MLP, RBF, SOM, and RF classifiers.

Classifier	LVQs		MLP		RBF		SOM		RF	
Datasets	Ave BR	Ave AR	BR	**AR**	BR	**AR**	BR	**AR**	BR	**AR**
Brain images data (PPUKM)	79.28	**85.20**	56.58	**67.76**	59.21	**71.05**	70.39	**75**	74.34	**80.92**
Segmented challenge (UCI)	88.45	**92.72**	88.67	**81.33**	88.67	**90.89**	75.78	**82.67**	92	**95.78**
Segment test (UCI)	88.27	**92.39**	67.49	**78.19**	85.19	**88.07**	72.02	**82.3**	88.48	**92.18**
Image segmentation (UCI)	90.33	**93.15**	77.2	**85.71**	85.14	**90.48**	77.34	**80.66**	91.92	**94.66**
Average and STD	86.58 ± 4.96	**90.87 ± 3.79**	72.49 **± **13.69	**78.25 ± 7.64**	79.55 ± 13.67	**85.12 ± 9.46**	73.88 ± 3.23	**80.16 ± 3.55**	86.69 ± 8.39	**90.89 ± 6.81**

(i) BR means before multirandomizing data sets.

(ii) AR means after multirandomizing data sets.

## Appendix

See Figure 12.
